# Accuracy and reliability of 3D cephalometric landmark detection with deep learning

**DOI:** 10.1186/s40001-025-03198-8

**Published:** 2025-10-21

**Authors:** Boyan Liu, Chang Liu, Yutao Xiong, Hailin Zhu, Wei Zeng, Jinglong Chen, Jixiang Guo, Wei Liu, Wei Tang

**Affiliations:** 1 State Key Laboratory of Oral Diseases & National Center for Stomatology & National Clinical Research Center for Oral Diseases & Department of Oral and Maxillofacial Surgery, West China Hospital of Stomatology, Sichuan University, No. 14, 3rd Section, Renmin South Road, Chengdu, 610041 People’s Republic of China; 2 Machine Intelligence Laboratory, College of Computer Science, Sichuan University, Chengdu, 610065 People’s Republic of China; 3 Foshan Stomatological Hospital, School of Medicine, Foshan University, Foshan, 528000 People’s Republic of China

**Keywords:** Craniofacial anatomy, Convolutional neural networks, Medical imaging, Landmark localisation

## Abstract

**Background:**

Three-dimensional (3D) landmark detection is essential for assessing craniofacial growth and planning surgeries, such as orthodontic, orthognathic, traumatic, and plastic procedures. This study aimed to develop an automatic 3D landmarking model for oral and maxillofacial regions and to validate its accuracy, robustness and generalizability in both spiral computed tomography (SCT, 41 landmarks) and cone-beam computed tomography (CBCT, 14 landmarks) scans.

**Methods:**

The model was implemented using an optimized lightweight 3D *U*-Net network architecture. Its accuracy, robustness and generalizability were thoroughly evaluated and validated through a multicenter retrospective diagnostic study. The model was trained and tested on a data set of 480 SCT and 240 CBCT cases. An additional inference on a different data set of 320 SCT and 150 CBCT cases was performed. Mean radial error (MRE) and success detection rate within 2-, 3-, and 4-mm error thresholds were measured as the primary evaluation metrics. Error analyses for landmark detection along each coordinate axis were performed. Consistency tests among observers were conducted.

**Results:**

The average MRE for both SCT and CBCT was consistently below 1.3 mm and, notably, below 1.4 mm in complex conditions, such as malocclusion, missing dental landmarks, and the presence of metal artifacts. No significant differences in MRE and SDR at 2–4 mm were observed between external and internal SCT and CBCT sets. SCT bone landmarks were more precise than dental ones, with no difference between bone/soft tissue and dental/soft tissue. CBCT dental landmarks exhibited greater precision compared to bone landmarks. A detailed error analysis across the coordinate axes showed that the coronal axis had the highest error rates. The implementation of this model significantly improved the landmarking proficiency of senior and junior specialists by 15.9% and 28.9%, respectively, while also achieving a 6–9.5-fold acceleration in GUI interaction time.

**Conclusions:**

This study shows that the AI-driven model delivers high-precision 3D localization of oral and maxillofacial landmarks, even in complex scenarios. The model demonstrates potential as a promising computer-aided tool to assist specialists in conducting accurate and efficient localization analyses; however, its robustness and generalizability require prospective clinical validation to ensure utility across varied experience levels.

**Supplementary Information:**

The online version contains supplementary material available at 10.1186/s40001-025-03198-8.

## Introduction

Accurate landmark identification is crucial for formulating treatment plans and evaluating surgical outcomes, as it significantly affects the precision of therapeutic interventions. In orthodontics and orthognathic surgery, landmarks are vital for cephalometric and aesthetic assessments [1–3]. In craniofacial trauma or reconstruction, comparing landmarks between unaffected and affected areas is essential for preoperative planning, intraoperative guidance, and postoperative evaluation [4, 5]. Clinicians typically use their expertise to manually or semi-automatically identify landmarks on lateral skull radiographs or computed tomography (CT) images, either incrementally on two-dimensional (2D) planes or using three-dimensional (3D) models for a holistic view. This process is laborious, time-consuming, and has a high learning curve, with considerable variability in outcomes due to subjective interpretation [1, 6].

Over the past decade, automated identification of craniofacial landmarks and derived analytical measurements has attracted considerable interest. Deep learning, particularly using convolutional neural networks (CNNs), has shown potential in surpassing traditional machine learning methods that depend on knowledge-based or atlas-guided techniques [7, 8]. Early studies focused on 2D lateral cephalograms [9], with a systematic review indicating a mean error of 1.39 mm for CNN-assisted automatic landmarking on 2D skull images, within a 95% confidence interval (CI) of 0.85–1.92 mm [10]. Despite the clinical use of deep learning software for 2D skull radiograph landmarking, 2D imaging has limitations, such as image superimposition, magnification issues, symmetry analysis complexities, and sensitivity to radiation exposure, projection angles, and patient positioning variations.

Unlike conventional 2D imaging, 3D imaging via CT scans overcomes the issue of image superimposition and offers a broad range of anatomical landmarks. Automated 2D craniometric landmark localization methods have achieved high accuracy, but training 3D landmark detection models faces greater challenges. Learning-based methods improve the detection accuracy by increasing the scale of model parameters and computational complexity. However, these methods also require substantial training data to ensure effective generalization and performance. This requirement is challenging to meet due to the scarcity of large public databases and the arduous, unreliable process of manual annotation [11]. A systematic review examining studies on automated landmark detection in 3D CT imaging [12] found significant methodological heterogeneity across research, with notable risks of bias in data selection and reference standards. Reported mean errors ranging from 1.0 to 5.8 mm may critically compromise the precision and robustness of cephalometric analysis data, highlighting the urgent need for more accurate automated detection algorithms.

As a result, research on automatic landmark detection in 3D CT imaging faces a few notable constraints. First, most studies relied on samples meticulously selected based on stringent and narrow inclusion criteria, which might fail to represent the full range of clinical scenarios [13, 14], such as diverse malocclusions, tooth loss, and metal artifacts. These limits have affected the validation of the accuracy and robustness of the models under various conditions. Second, while specialized dental hospitals often use cone-beam computed tomography (CBCT) scans, general hospitals prefer spiral computed tomography (SCT) scans due to its advantages in soft tissue visualization, despite higher radiation and lower spatial resolution. SCT is widely used for craniofacial diagnostics and treatments, including aesthetic surgery; however, there is a lack of comparative studies on the performance of automatic landmarking models between these imaging modalities.

This study developed a streamlined, lightweight 3D *U*-Net network for automatically detecting oral and maxillofacial landmarks in SCT and CBCT images. Rigorous validation confirmed its reliability, robustness, and generalizability. This deep learning application, tested across a multicenter data set, aims to meet clinical requirements for accurate 3D cranial landmarking. It is designed to enhance specialists’ accuracy, lessen the burden of manual annotation, and emulate senior clinicians’ expertise.

## Materials and methods

This study was conducted in West China Hospital of Stomatology, Sichuan University, following Declaration of Helsinki, the ethical guidelines of the International Committee of Medical Journal Editors, Schwendicke et al. checklist for artificial intelligence in dental research [15], and the Transparent Reporting of a multivariable prediction model for Individual Prognosis or Diagnosis (TRIPOD) statement [16]. This study was approved by the Institutional Review Board of West China Hospital of Stomatology, Sichuan University (ethical approval No.: WCHSIRB-D-2023-011).

### Data collection

The internal data set was retrospectively collected from the Department of Radiology at West China Hospital of Stomatology from September 2020 to October 2022. The external data sets were derived from Tianfu Hospital and Jinjiang Outpatient Clinic between November 2022 and December 2023. A consecutive sampling method was used, and the information was anonymized using numerical codes.

Inclusion criteria were as follows: (1) SCT and wide-field CBCT images of patients aged 6–70 years; and (2) for multiple imaging sessions, only the first set was used. The exclusion criteria were as follows: (1) low resolution, high noise, motion artifacts, or incomplete field of view, which hinder accurate landmark identification; and (2) patients with malignant tumors, fractures, or post-surgery conditions. However, subjects with orthodontic appliances or metal osteosynthesis plates were not excluded, provided these metallic implants did not obstruct landmark recognition.

### Landmark selection and definition

As detailed in Supplementary Table A1, the SCT data set comprised 41 craniometric landmarks: 21 midline singular landmarks, 10 bilateral pairs (denoted L/R for left/right). Concurrently, the CBCT osteodental analysis targeted 14 diagnostically critical markers, including 8 skeletal midline references (Nasion [N], Sella [S], Anterior Nasal Spine [ANS], Subspinale [A], Supramentale [B], Pogonion [Pog], Menton [Me], Gnathion [Gn]), 6 bilateral dental landmarks (UI, LI, U6L/R, L6L/R). The difference in the number of landmarks between SCT and CBCT data sets reflected their distinct clinical applications. SCT was typically used for complex craniofacial assessments, necessitating a larger set of landmarks to capture the full anatomical complexity. In contrast, CBCT was predominantly used for dental and maxillofacial applications, where fewer, more specialized landmarks were sufficient to address the specific clinical questions related to dental and jaw structures. This distinction ensured that each modality’s landmark set was optimized for its intended diagnostic purpose.

### Data annotation and reference standard

The SCT images were independently annotated by a senior oral and maxillofacial surgeon (BY. L., 9 years of experience), while CBCT annotations were performed by a senior orthodontist (Ch. L.; 14 years of experience). All landmark delineations underwent rigorous quality control by a chief physician (W. T., 31 years of experience in dental imaging). Image processing and 3D reconstruction were executed using Mimics 16.0 (Materialize Interactive Medical Image Control System, Belgium). Thresholds were set for bone (226–2619 HU) and soft tissue (− 700–225 HU) in SCT and a specific range (720 + HU) for CBCT to generate 3D models. In the software’s “Measurement and Analysis” module, a custom landmark annotation tool was created. Two reference observers marked these sequentially, starting with the approximate positions of the 3D model. The location of the midline landmarks was refined on the sagittal images to align with the tissue surface. For paired bone landmarks, the horizontal images were adjusted to match the tissue surfaces. Dental landmarks were positioned at the tooth apices using both sagittal and horizontal CT images. Reviewers adjusted following the same order. The positional data of the points were stored in eXtensible Markup Language (XML) format.

Before annotation, training was conducted to ensure consistency among annotators, with the aim of standardizing the use of software tools for key landmark annotation. Two annotators labeled 50 images, recording landmark coordinates along the *x*-(axial), *y*-(sagittal), and *z*-(coronal) axes. After a 4-week re-annotation for reliability assessment, landmarks with an intraclass correlation coefficient (ICC) ≥ 0.70 were set as the “reference standard”. It took 10–14 min to annotate each case.

### Model establishment

**Fig. 1:**
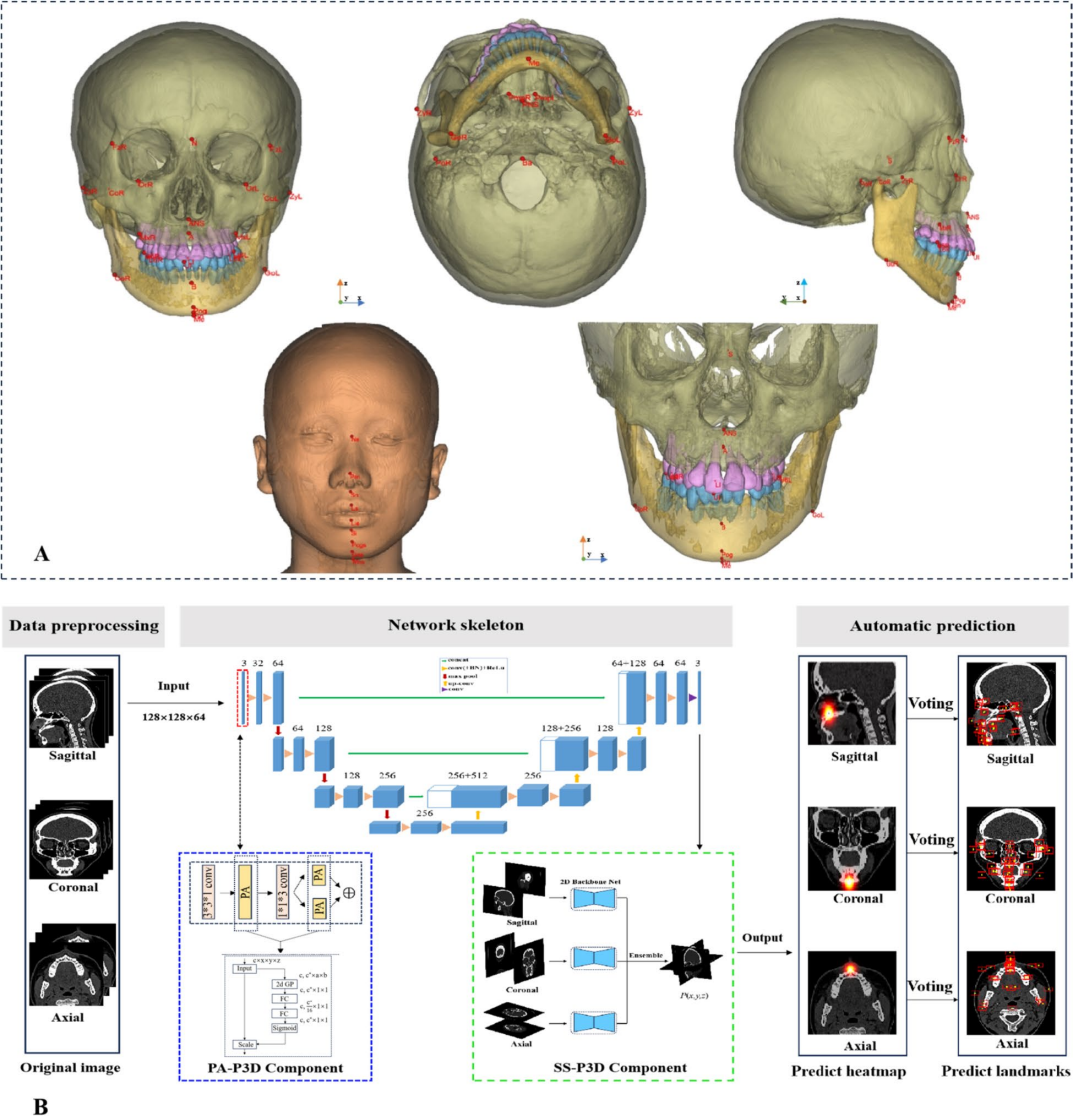
Schematic illustration of the automatic landmarking procedure utilizing the enhanced 3D U-Net model. **A** First row (left to right): SCT anterior, superior, and lateral views; second row (left to right): soft tissue and CBCT landmarks; 41 landmarks marked in red: 26 bony, six dental, and nine soft tissues. **B** Specifically, the 3 × 3 × 3 convolution (red dashed frame) in each downsampling stage was replaced by the PA-P3D block (blue dashed frame). The general P3D block replaced the 3D convolution with two spatiotemporal substantial convolutions, thereby reducing parameters (from n^3^ to n^2^ + n). This approach resulted in a significant reduction in the model’s parameter count and computational cost. Following the same process as that used by clinical doctors to manually localize landmarks through three planes, a novel Spatial Separation Pseudo-3D (SS-P3D) model transformed the 3D landmark detection task into three separate 2D landmark detection tasks (green dashed frame). Each branch of the SS-P3D model used an encoder–decoder structure to transform the input into a same-size feature map. Subsequently, the general Gaussian heatmap regression was applied to locate the 2D landmarks in the corresponding slice. Finally, the coordinates of these candidate landmarks were averaged to obtain the final prediction

The hardware environment for this experiment was as follows: CPU, Intel^®^ Core^™^ i5-12600KF Processor (20 M Cache, up to 4.90 GHz); GPU, Nvidia GeForce RTX^™^ 3080 Ti; and Memory, 32 GB. The software environment was as follows: operating system, Ubuntu 20.04.1; and Programming Language, Python 3.8.10. The streamlined, lightweight 3D U-Net network adopted the strategy of constructing planewise attention pseudo-3D (PA-P3D) blocks to optimize the parameters and integrated spatial separation pseudo-3D (SS-P3D) models for precise localization (Fig. 1). In the Appendix, the comparative baseline models including *V*-Net, FC-DenseNet, and Hourglass, were implemented under controlled conditions.

The SCT and CBCT images from the internal data set were used to develop this model, with a ratio of training set:validation set:test set = 7:1:2. The model was implemented using PyTorch. As demonstrated in Supplementary Figure A1, consistent hyperparameter configurations were maintained across both SCT and CBCT modalities, including a stochastic gradient descent with a momentum of 0.9, learning rate of 0.001, weight decay of 0.0005, and learning rate reduction of 0.1 every 50 epochs. Training would end after 60 epochs if the validation loss did not decrease further for 20 epochs, or after 150 epochs. The same hyperparameters were applied to SCT and CBCT, with the CBCT model weights optimized for stable loss.

### Quantitative evaluation of external data sets

To evaluate the generalization of the deep learning model on new data, an external test set of SCT and CBCT images from different centers was used. This approach was further utilized to evaluate the model’s robustness in the face of challenges, such as malocclusion, missing dental landmarks, and metal artifacts. To identify the sources of error, the deviations in landmark localization were analyzed across each coordinate axis.

### Clinical evaluation observers assessing AI system performance

The dual-phase evaluation protocol was designed to quantify the AI system’s clinical utility. In phase 1 (Baseline Assessment), two oral and maxillofacial radiologists (junior: 5 years; senior: 11 year experience), serving as clinical evaluation observers independently annotated 40 external cases (20 SCT/20 CBCT). Following a 1-month washout period, Phase 2 (AI-assisted reevaluation) reintroduced the same cases. In this phase, AI-predicted landmarks were overlaid at 50% transparency. Observers used a graphical user interface (GUI) to either accept the markings or manually adjust them using a cursor, with the time-per-case recorded via an embedded chronometry module. The Manual Refinement Protocol required adjustments to be made using spline-calibrated cursors for 0.1 mm precision control, coupled with multi-planar synchronization to maintain spatial consistency across axial, sagittal, and coronal views.

Due to sample size limitations and restricted subject enrollment, we implemented three key measures to mitigate potential biases: (1) randomized case presentation sequences during each evaluation session to counteract recall bias, (2) complete separation of clinical evaluators from reference standard establishment ensuring independent assessment, and (3) imposition of no time constraints during clinical evaluations to enable comprehensive analysis and minimize procedural pressure effects.

Statistical analysis encompassed three domains: intra-rater consistency assessed through Bland–Altman plots and a novel within-observer variability index (WOVI) derived from Hausdorff distance (95% CI); inter-modality agreement evaluated via two-way mixed ICC (3,1) models stratified by imaging modality.

### Evaluation metrics

This study employed the mean radial error (MRE) and success detection rate (SDR) within the error thresholds of 2, 3, and 4 mm as key evaluation metrics [17]. Landmark identification precision was compared across complex scenarios and different tissues and within distinct anatomical areas. The consistency within observers and that between observers and the AI model were evaluated using ICC. Notably, the term “observers” in this study encompasses both the reference observers and clinical evaluation observers.

In cases where the model identified a dental landmark that was actually missing or not present in the image, the data from these incorrectly identified landmarks were excluded from the calculations of MRE and SDR. This exclusion ensures that the evaluation metrics reflect the model’s performance on valid, correctly identifiable landmarks. The Dental Landmark Accuracy (DL-ACC) and Dental Landmark Precision (DL-PRE) metrics further assess the model’s capability to detect both the presence and accurate positioning of dental landmarks, ensuring a comprehensive evaluation of its diagnostic consistency and efficiency. All metrics are detailed in the Appendix.

### Statistical analysis

Statistical analyses were performed using IBM SPSS software (version 22; IBM Corp. Armonk, N.Y., USA) with a significant level (*α*) of 0.05. Quantitative data (e.g., age and MRE) are presented as means and standard deviations (SD). Moreover, 95% confidence intervals (CI) were calculated for MRE. All quantitative data—including mean radial errors (MRE)—were analyzed exclusively with non-parametric methods due to the right-skewed distribution of errors bounded at zero. Specifically, two-group comparisons (e.g., overall errors between SCT and CBCT modalities) employed the Mann–Whitney *U* test; multi-group comparisons (such as error analysis across anatomical regions) utilized the Kruskal–Wallis *H* test; subsequent post-hoc pairwise analyses following significant Kruskal–Wallis *H* test results implemented the Nemenyi test, while categorical variables (including demographic parameters like sex distribution) were analyzed using Pearson’s chi-squared test.

### Sample size calculation in testing data set

The target sample size of the testing set was at least 68 samples to detect a difference of 0.4 mm in MRE from the reference baseline (1.5 mm), assuming a significant level of 0.05 (type I error *α* = 0.05, two-sided), 90% power (type II error *β* = 0.1), and a standard deviation of 1 mm. The total summed number of either SCT or CBCT images in both the internal and external testing sets should be not less than 68.

## Results

### Sample source and baseline data

The baseline demographic and clinical characteristics of the data set as well as the equipment used are detailed in Table 1. The samples were derived from 800 SCT data and 390 CBCT data cases across three centers. The average age of the patients from three centers was 30.41 ± 8.65 years (range 6.5–69.5 years), with a Kruskal–Wallis *H* test indicating no significant difference across three centers (*P* = 0.324). The male-to-female ratio was 621:569 (52.2% male), and a chi-square test showed no significant difference in sex distribution (*P* = 0.874).

**Table 1:** Data set characteristics

	*N* (male/female)	Age (Y ± SD)	Equipment^*^	*M*	MDL	MA
SCT internal data set (Center 1)	480 (265/215)	28.03 ± 8.58	Philips MX 16 CT	216	148	78
CBCT internal data set (Center 1)	240 (117/123)	33.20 ± 7.09	1. Morita 3D Accuitomo 2. Planmeca ProMax 3D 3. LARGEV HiRes	102	48	27
SCT external test set (Center 2)	320 (172/148)	34.57 ± 8.13	Philips Brilliance 64 GE Discovery CT750	104	64	28
CBCT external test set (Center 3)	150 (67/83)	24.65 ± 6.02	1. NewTom VGi 2. LARGEV Smart3D	36	54	53
Total	1190 (621/569)	30.41 ± 8.65		458	314	186

*n* number of cases, *M* malocclusion, *MDL* missing dental landmarks, and *MA* metal artifacts

Center 1: Stomatology Hospital. Center 2: General Hospital Radiology. Center 3: Dental Clinic for Malocclusion

*Three SCT types: 0.32–1.0-mm slice interval, 0.625–1.0-mm thickness, and 0.45-mm (0.32–0.63 mm) pixel size. Matrix: 512^3^. Five CBCT types: 70–120 kV, 3–14 mAs, 0.25-mm (0.15–0.40 mm) voxel size. Matrix: 610–670^3^

### Performance of improved 3D U-Net model

Table 2 details the modified 3D *U*-Net performance on both test sets. No significant MRE difference was found between external and internal SCT sets (Mann–Whitney *U* test, *P* = 0.11), and no significant SDR difference at 2, 3, and 4 mm (*P* = 0.13, 0.26, 0.62). Similarly, no significant MRE difference was found between external and internal CBCT sets (Mann–Whitney *U* test, *P* = 0.41), and no significant SDR difference at 2, 3, and 4 mm (*P* = 0.08, 0.36, 1.00).

**Table 2:** Automatic skull landmarking based on improved 3D *U*-Net (*n* = number of cases)

Data set (*n*)	Types	MRE ± SD, mm	MRE, 95% CI, mm	SDR, %		
				2 mm	3 mm	4 mm
Internal test set (SCT: 96; CBCT: 48)	SCT	1.091 ± 0.306	1.037–1.145	92.6	99.1	99.7
	CBCT	0.924 ± 0.830	0.819–1.029	99.1	100	100
SCT external test set (*n* = 64)	Normal	1.217 ± 0.615	1.109–1.325	91.6	99.1	100
	M	1.228 ± 0.832	1.068–1.388	88.3	98.9	98.9
	MDL	1.225 ± 0.710	1.051–1.399	90.0	99.1	99.1
	MA	1.240 ± 0.566	1.030–1.450	88.3	97.1	99.1
	Average	1.224 ± 0.720	1.146–1.302	90.5	98.9	99.4
CBCT external test set (*n* = 30)	Normal	0.916 ± 0.798	0.444–1.388	99.1	100	100
	M	1.115 ± 0.882	0.683–1.547	94.9	99.1	100
	MDL	0.966 ± 0.844	0.648–1.284	99.1	100	100
	MA	1.082 ± 0.756	0.686–1.478	99.1	100	100
	Average	1.012 ± 0.530	0.854–1.170	93.2	98.4	100

*M* malocclusion, *MDL* missing dental landmarks, *MA* metal artifacts, *MRE* mean radial error, *CI* confidence Interval, and *SDR* successful detection rate

### Landmark detection in SCT and CBCT: varying conditions, tissues, regions

In the external SCT and CBCT data sets presented in Table 2, Kruskal–Wallis *H* tests were used to compare the MREs of landmarks in samples with malocclusion, missing dental landmarks, and metal artifacts compared to normal samples (SCT: *P* = 0.265; and CBCT: *P* = 0.135), showing no statistical difference.

**Fig. 2:**
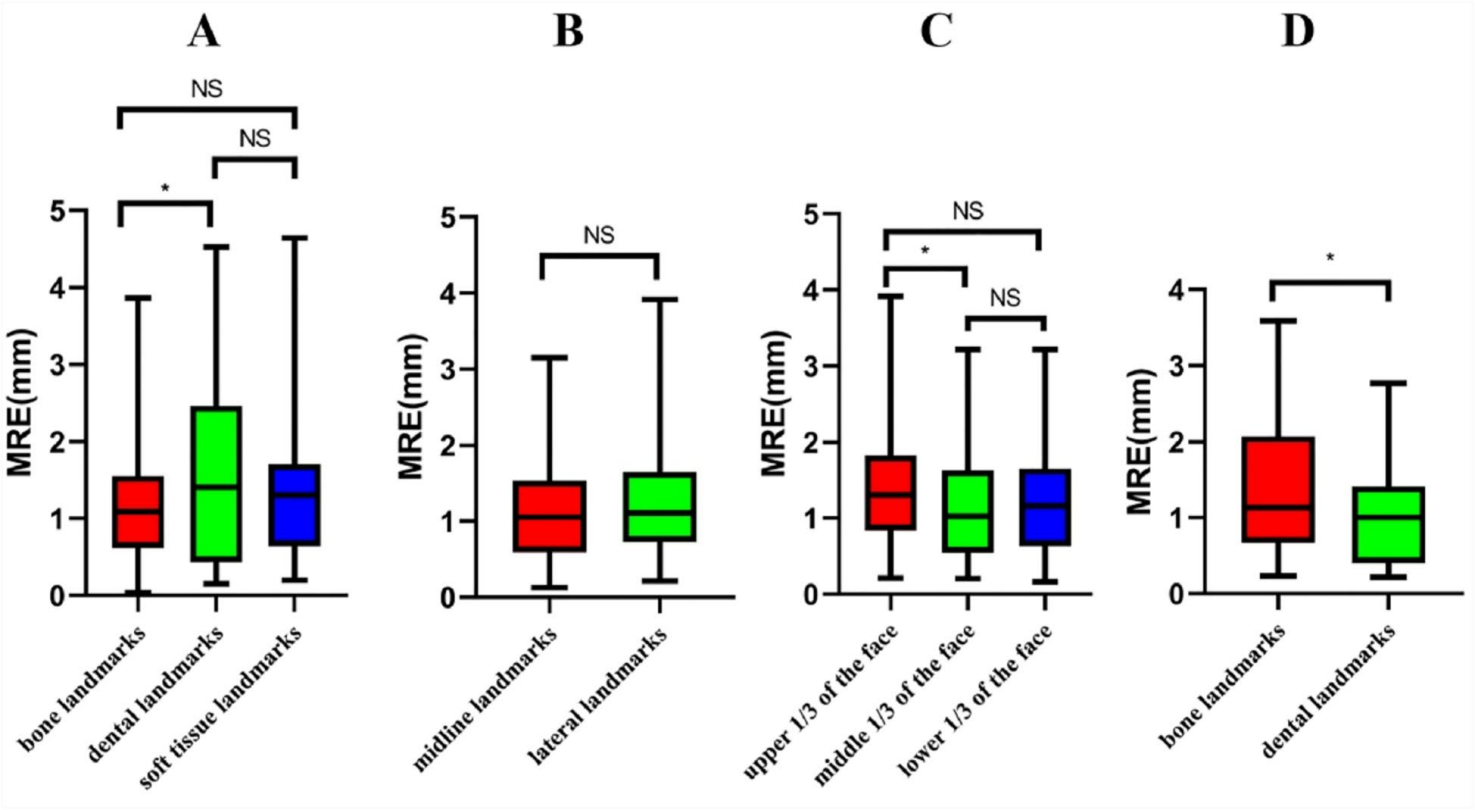
Box plots comparing MRE across different SCT and CBCT landmarks. **A** SCT bone, soft tissue, and dental landmarks; **B** SCT midline and lateral landmarks; **C** SCT upper ⅓, middle ⅓, and lower ⅓ landmarks; and **D** CBCT bone and dental landmarks. (*: *P* ≤ 0.05, NS: *P* > 0.05)

In Fig. 2, box plots were used to compare MRE across different SCT and CBCT landmarks. SCT bone landmarks were more accurate than dental landmarks (Nemenyi test, *P* = 0.021), with no difference between bone and soft tissue or dental and soft tissue. No MRE difference was found between SCT midline and lateral landmarks. SCT middle ⅓ landmarks were more accurate than upper ⅓ landmarks (Nemenyi test, *P* = 0.043), with no difference between upper ⅓ and lower ⅓landmarks or middle ⅓ and lower ⅓landmarks. CBCT dental landmarks were more accurate than bone landmarks (Mann–Whitney *U* test, *P* = 0.032). All SCT and CBCT landmark measurements were listed in the Appendix.

### MRE in missing dental landmark images

**Fig. 3:**
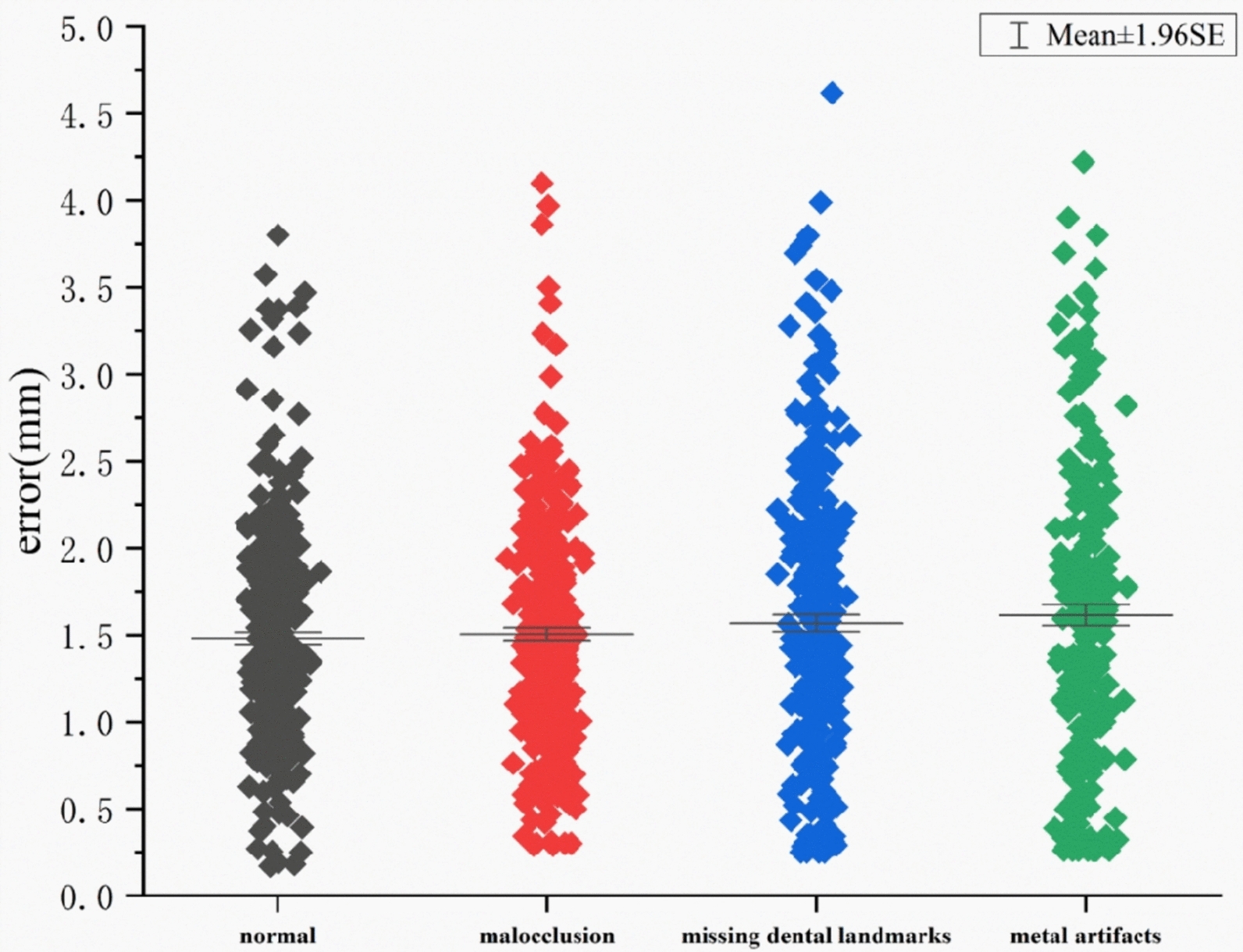
Scatter plot of MRE dental landmarks in the external test set created with Origin 2022. The MRE distributions among normal, malocclusion, missing landmarks, and metal artifacts were similar, showing no significant differences (Kruskal–Wallis *H* test, *P* = 0.105)

Six dental landmarks were identified. DL-ACC and DL-PRE evaluated landmarking in images with missing dental landmarks, achieving 97.73% and 96.98% accuracy, respectively. The scatter plot in Fig. 3 showed the accuracy of the model in detecting the presence or absence of dental landmarks. The exclusion of anatomically absent dental landmarks from MRE/SDR calculations could optimistically bias performance estimates. However, the complementary DL-ACC/DL-PRE metrics confirmed robustness. Clinically, this suggested the model rarely hallucinates landmarks in edentulous regions.

### Landmark positioning errors along each coordinate axis

**Fig. 4:**
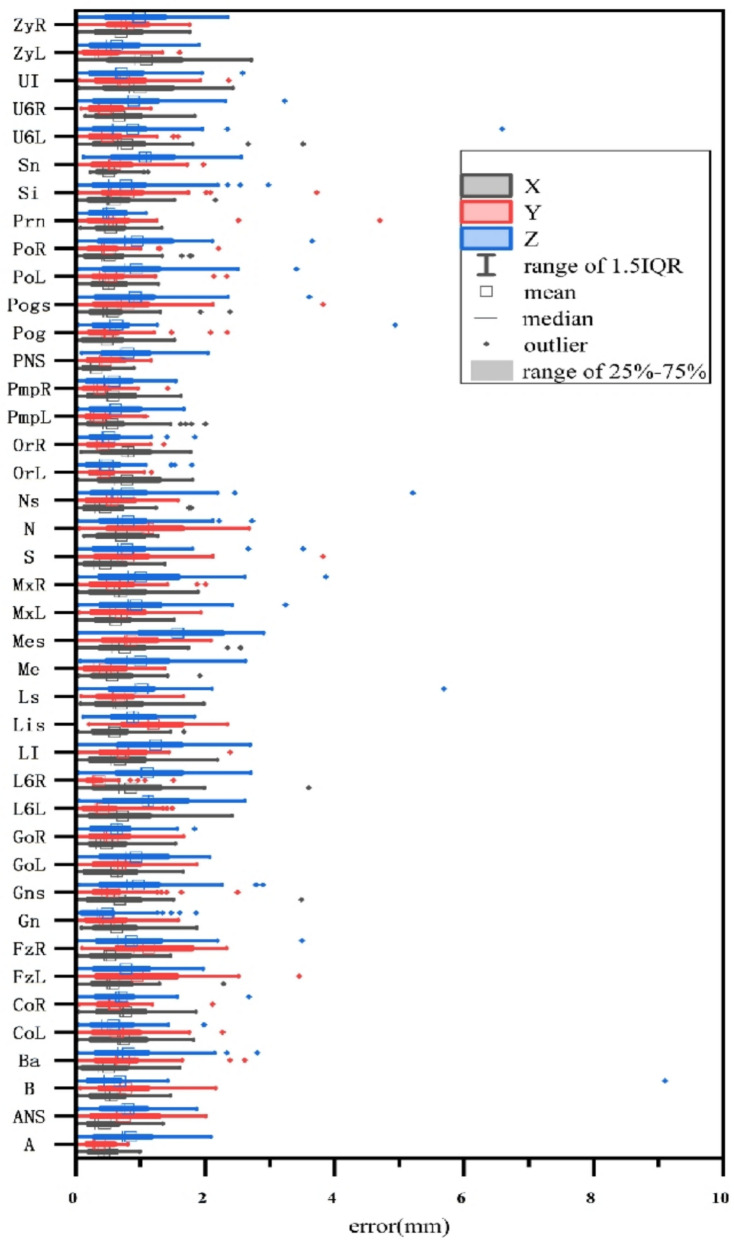
Box plot of landmark localization errors by group and axis

Figure 4 illustrates the landmark localization errors along each coordinate axis for the test set. The mean radial error (MRE) was 0.81 ± 0.23 mm (range: 0.11–1.97 mm) for the axial (*x*-axis), 0.81 ± 0.69 mm (range: 0.17–2.20 mm) for the sagittal (*y*-axis), and 1.14 ± 0.30 mm (range: 0.13–2.75 mm) for the coronal (*z*-axis). Among the 41 landmarks analyzed, the largest positioning errors were predominantly observed on the *z*-axis, with 26 landmarks (63.4%) showing higher errors compared to 7 landmarks (17.1%) on the *x*-axis and 8 landmarks (19.5%) on the *y*-axis. The PNS landmark on the *x*-axis exhibited the lowest error (MRE: 0.804 ± 0.509 mm, mean △x: 0.21 ± 0.10 mm), while the Mes landmark on the *z*-axis had the highest error (MRE: 1.714 ± 0.989 mm, mean △z: 1.49 ± 1.26 mm). Notably, the errors for L6R on the *y*-axis and Pogs on the *x*-axis exceeded the 75th percentile, indicating potential outliers. The remaining 39 landmarks showed average errors within the 25th to 75th percentile range.

High-error landmarks across modalities were identified and summarized in Supplementary Tables A6–A7. In SCT, LI showed the highest MRE (1.745 ± 0.849 mm), driven largely by the *z*-axis deviation (Δz = 1.39 ± 1.02 mm). In CBCT, GoR reached an MRE of 1.491 ± 0.741 mm with errors dispersed across all axes (Δx = 0.29 ± 0.26 mm; Δy = 0.37 ± 0.31 mm; Δz = 1.19 ± 0.97 mm).

### AI system versus clinical observers: diagnostic consistency and efficiency comparison

Table 3 shows a comparison of the AI system’s diagnostic consistency with two clinical evaluation observers. Differences were observed in performance between junior and senior radiologists, with MREs of 1.895 and 1.325 (Mann–Whitney *U* test, *P* = 0.013), and DL-ACCs of 95.12% and 98.54% (Mann–Whitney *U* test, *P* = 0.038), respectively. The AI model performed similar to the senior specialist, with MREs of 1.246 and 1.325, respectively (Mann–Whitney *U* test, *P* = 0.16), and reduced the GUI landmarking time by a factor of 6–9.5. Both manual and AI methods had excellent ICC values, with over 90% of landmarks scoring > 0.9.

**Table 3:** Assessment of clinical evaluation observers–AI landmarking consistency trial (mean ± SD)

	MRE ± SD	DL-ACC	DL-PRE	ICC	Time (min)
AI	1.246 ± 0.71^a^	97.86%^c^	97.12%^e^	1	1/30
Junior specialist	1.895 ± 0.85^a, b^	95.12%^c,d^	92.63%^e,f^	0.932	19 ± 2.1^ g^
Junior specialist + AI	1.347 ± 0.62	96.12%	94.62%	0.967	2 ± 0.8^ g^
Senior specialist	1.325 ± 0.45^b^	98.54%^d^	97.45%^f^	0.976	12 ± 1.6^ h^
Senior specialis + AI	1.114 ± 0.53	99.53%	99.89%	0.991	2 ± 0.7^ h^

Values in the same column that share the same superscript letter indicate a significant difference (*p* < 0.05)

**Table 4:** Comparison of CT landmarking with state-of-the-art research

		This study	Dot research
Sample size		1190 (800 SCT, 390 CBCT)	198 (SCT)
Data source		3 centers, 3 SCT types 5 CBCT types	1 center, 5 SCT types
Sample Characteristics	M	Present/analyzed	Present/analyzed
	MDL	Present/analyzed	Not mentioned/not analyzed
	MA	Present/analyzed	Present/not analyzed
Number of landmarks		41 (SCT), 14 (CBCT)	33 (SCT)
MRE ± SD (mm)		1.22 ± 0.72 (SCT) 1.012 ± 0.530 (CBCT)	1.0 ± 1.3 (SCT)
SDR_2mm_		90.5% (SCT) 93.3% (CBCT)	90.4% (SCT)
SDR_3mm_		98.9% (SCT) 98.3% (CBCT)	95.4% (SCT)

*n* number of cases, *M* malocclusion, *MDL* missing dental landmarks, *MA* metal artifacts, *MRE* mean radial error, *SD* standard deviation, *SDR* success detection rate

### 3D visualization presentation

**Fig. 5:**
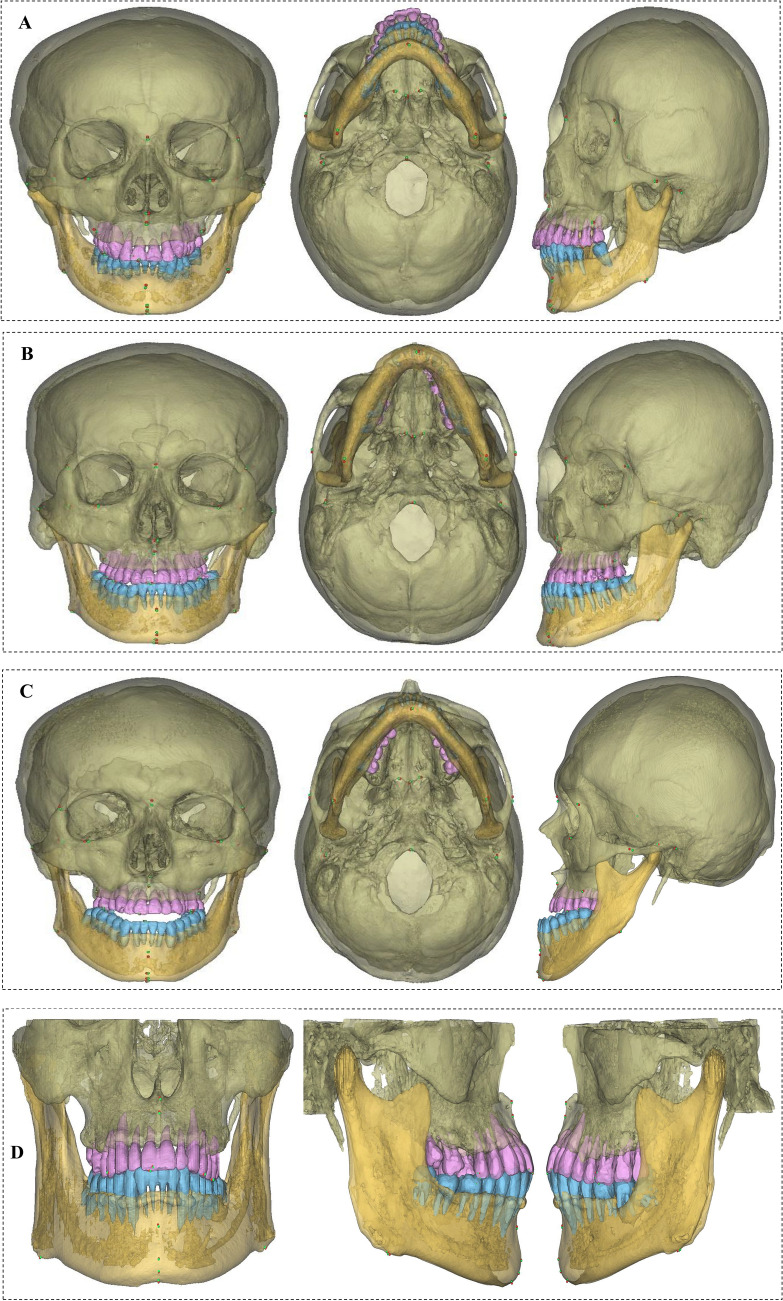

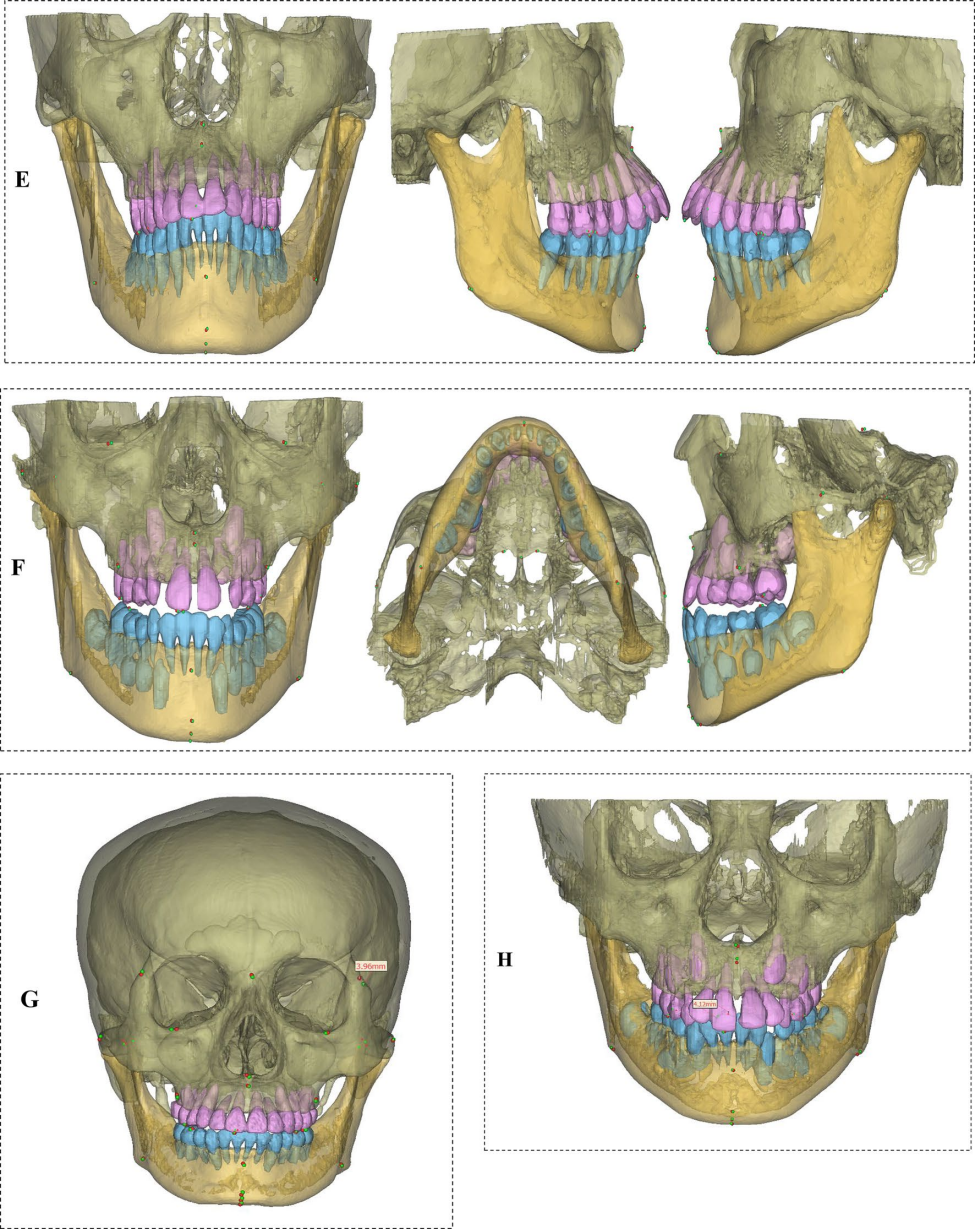
Frontal, bottom, and left views of the three-dimensional models of SCT and CBCT. Red landmarks represent the reference standard, and green ones are the predicted landmarks. *M* malocclusion, *MDL* missing dental landmarks, *MA* metal artifacts. **A (MDL):** SCT sample with the first molar on the left side of the mandible missing, where the L6L landmark was absent, with an MRE of 1.41 mm for all displayed landmarks. **B (M + MA):** In this SCT sample exhibiting crossbite, a porcelain crown was present on the left upper premolar. The landmarks primarily impacted by these artifacts were the UI and LI, with localization errors of 1.24 mm and 1.26 mm, respectively. **C (M):** SCT malocclusion sample, with an MRE of 1.34 mm for all displayed landmarks. **D (MDL):** CBCT sample with tooth absence, featuring a landmark MRE of 0.98 mm. **E (M):** Skeletal malocclusion CBCT, with a landmark MRE of 1.01 mm. **F (MDL):** A CBCT sample from a 7-year-old child with tooth absence, featuring a landmark MRE of 0.94 mm. **G (M):** One case of malocclusion, with an average MRE of 1.47 mm on SCT. The largest error was at the FzL point (The most medial and anterior point of each frontozygomatic suture at the left orbital rim), with an error of 3.96 mm, which is the landmark with the largest error among all SCTs. **H (M + MDL):** One case of malocclusion with tooth loss in an 8-year-old child, with a landmark MRE of 1.18 mm on CBCT. The largest error was at the LI point (The most midpoint of the crown tip of the right lower central incisor), with an error of 4.12 mm, which is the landmark with the largest error among all CBCTs

To fully demonstrate the performance of this model, representative cases from the external test set were analyzed (Fig. 5a–h). Additional cases, including those involving soft tissue localization and a case where SCT and CBCT were acquired at different timepoints, are presented in the Appendix.

### Comparison of related studies

## Discussion

High-precision, automated 3D landmarking technology holds significant potential for application in the field of oral and maxillofacial surgery. Current automated detection methods in 3D CT imaging face issues, such as large parameter scales, high computational complexity, and data annotation difficulties. In addition, they lack robust validation across complex clinical scenarios (e.g., malocclusion, missing teeth, and metal artifacts) and different imaging modalities (SCT vs. CBCT) [11, 12]. To bridge these gaps, we designed a multi-center study that incorporates both SCT and CBCT with clinically challenging phenotypes (Table 4).

Compared with the benchmark research (single-centre, 198 SCT images, MRE 1.0 ± 1.3 mm) [18], the present multicentre data set (1190 scans, MRE 1.22 ± 0.72 mm) achieves comparable precision while improving the 3 mm success detection rate (98.9% vs. 95.4%). The inclusion of CBCT (MRE 1.01 ± 0.53 mm) and the explicit modelling of malocclusion, missing dentition and metal artifacts extend the robustness assessment to clinical scenarios that have been sparsely documented.

Unlike other studies that include both SCT and CBCT data sets [19–21], this study shares hyperparameters between SCT and CBCT models, with their weight parameters determined through training. This study found that SCT’s MRE for bone landmarks is lower than that for dental landmarks, while CBCT shows the opposite trend, with both differences being statistically significant. Dental landmarks in spiral CT have a higher MRE than average; however, in CBCT, they have a lower MRE, likely due to the better spatial resolution and dental imaging capabilities of CBCT [22]. Previous studies have noted modality-specific challenges in cephalometric analysis [23]. In this study, SCT utilized a pixel size of 0.32–0.63 mm (matrix 512^3^), while CBCT employed a voxel size range of 0.15–0.40 mm (matrix 610–670^3^). The inhomogeneity in CT image quality significantly impacts image resolution and anatomical contrast, which directly contributes to measurement errors and is manifested by higher standard deviations in MRE (as shown in Table 2). This indicates that higher resolution CT modalities provide more accurate and stable outcomes. These findings underscore the necessity of standardized clinical imaging protocols to reduce variability.

The model localization strategy is key to system performance. Lightweight models such as *V*-Net or 3D *U*-Net are popular but may require reduced image resolution, affecting accuracy. A common solution is a multi-stage, coarse-to-fine approach [24–26]; however, adding 3D CNNs can boost the parameter count and deployment costs, with some models taking up to 83 s [27]. This study presents a novel pseudo-3D architecture that transitions from coarse to fine with the PA-P3D and SS-P3D models, maintaining global–local dependencies despite potential detail loss from downsampling [27]. Hence, this study tested the 3D *U*-Net architecture against the *V*-Net [28], FC-DenseNet [29], and Hourglass [30] networks (detailed in the Appendix). The enhanced 3D *U*-Net demonstrated a superior accurate localization performance compared to that of other network structures, with an average processing time of less than 2 s per case (GPU: Nvidia GeForce RTX^™^ 3080), thus offering a novel solution for medical image localization with varying resolutions.

Understanding the distribution of errors is crucial for anticipating potential measurement bias, as demonstrated in previous studies [10, 11]. The present investigation reveals that the *z* (coronal)-axis exhibits the highest frequency of landmarks with the most substantial errors. This finding suggests that the slice thickness significantly influences the error in AI-based landmark identification; enhanced precision is achieved with thinner slices or CT images of higher resolution. To elucidate the anatomical–imaging basis of the observed *z*-axis dominance, we interrogated the highest error landmarks. In the context of manual landmarking, those defined by intricate anatomical curves or specific directional projections tend to exhibit greater errors, necessitating ongoing training and practice for readers to enhance the accuracy and consistency of landmark localization [15, 31].

Given that *z*-axis error predominates, we next examined which anatomical landmarks are most susceptible to slice-thickness-related inaccuracies. Associations between high-error landmarks and their anatomical–imaging characteristics were evident. The LI exhibited pronounced error, likely linked to its variable crown morphology and susceptibility to occlusion artifacts. The GoR showed elevated uncertainty in CBCT, attributable to blurred margins and irregular cortical contours at the mandibular angle. Inspection of Fig. 5g, h reveals maximal deviations for FzL in SCT and LI in CBCT. Probable contributors include (i) an undersized crop (128 × 128 × 64) that incompletely encompasses the zygomatic complex for boundary-dependent points, such as FzL, and (ii) a *σ* = 20 Gaussian kernel that may over-smooth millimetric shifts characteristic of landmarks like LI, mandating re-evaluation of the augmentation pipeline. Future work will implement an alert system for error-prone regions that triggers mandatory human review.

Having characterized error patterns, we next examined whether AI could consistently outperform human observers under controlled conditions. The study included assessments of intra- and inter-observer consistency and reliability to evaluate the AI model’s performance. Our analyses indicate that, under identical experimental conditions, AI demonstrated accuracy comparable to senior specialists and significantly higher than junior specialists. However, due to limitations in the observer trial design—including a small sample size (*n* = 40), lack of blinding, and potential halo effects—these results should be interpreted with caution and cannot fully substantiate clinical reliability. This suggests the potential of AI as an assistive computer-aided tool for landmark identification, particularly in augmenting less experienced clinicians. Nevertheless, rigorous prospective validation with larger cohorts and randomized designs is essential before considering clinical adoption.

Addressing these limitations will require multi-institutional prospective trials, larger age-span cohorts, and integration of explainable AI frameworks. Although the data set included images of varying sizes and resolutions from SCT and CBCT, as well as special types, such as deformities, tooth loss, and metal artifacts, and samples across a certain age range, the data set still faced issues with limited diversity. Further research should evaluate the impact of landmarking errors on widely used linear and angular measurements, further improve the reference standard precision of landmarks on the *z* (coronal)-coordinate, and select high-precision, high-reliability landmarks among multiple landmarks in local areas. It is worth noting that AI processes information quickly; however, its accuracy does not surpass that of experts, and acceptable error margins vary depending on the treatment type. This underscores the imperative for integrating explainable AI (XAI) methodologies and ethical safeguard mechanisms, such as those advocated by Thurzo et al. [32, 33], to establish clinician-trustworthy workflows before deploying AI-driven landmarking in clinical practice.

## Conclusion

This study used an optimized lightweight 3D *U*-Net architecture to develop an automatic localization model for SCT and CBCT imaging. The model achieved high accuracy in the 3D localization of oral and maxillofacial structures, even in complex cases with precision less than 1.5 mm. Designed as a promising computer-aided tool, this model may assist specialists in performing precise and efficient localization analyses; however, its robustness, generalizability, and clinical utility across diverse experience levels require prospective validation in real-world clinical settings. We will focus on improving the transparency and safety of the model in the future, particularly in influencing personalized treatment decisions across various medical fields. We envision this model as a valuable clinical tool, but emphasize the need for a cautious and standardized clinical evaluation to ensure its broad effectiveness.

## Supplementary Information

Additional file 1.

## Data Availability

No datasets were generated or analysed during the current study.

## Abbreviations

CT: Computed tomography
SCT: Spiral computed tomography
CBCT: Cone beam computed tomography
3D: Three-dimensional
2D: Two-dimensional
MRE: Mean radial error
SDR: Success detection rate
DL-ACC: Dental Landmark Accuracy
DL-PRE: Dental Landmark Precision
ICC: Intraclass correlation coefficient
M: Malocclusion
MDL: Missing dental landmarks
MA: Metal artifacts
