# Supplementary material for: Accuracy and reliability of 3D cephalometric landmark detection with deep learning
Source: Eur J Med Res. 2025 Oct 21;30:1000. doi: 10.1186/s40001-025-03198-8 (PMC12539169; doi:10.1186/s40001-025-03198-8)
Supplement: Supplementary file 1 — Additional file 1. [file 40001_2025_3198_MOESM1_ESM.docx]

**Accuracy and Reliability of 3D Cephalometric Landmark Detection**

**with Deep Learning**

Boyan Liu, Chang Liu, Yutao Xiong, Hailin Zhu, Wei Zeng, Jinglong Chen, Jixiang Guo, Wei Liu, Wei Tang

**Supplemental Appendix**

**1 Materials and Methods**

The spiral computed tomography (SCT) dataset comprises 41 anatomically validated landmarks, systematically stratified into 21 unilateral reference points and 10 bilateral pairs (denoted L/R for left/right). In parallel, the cone-beam computed tomography (CBCT) analysis incorporates 14 osteodental landmarks: eight skeletal midline markers (N, S, ANS, A, B, Pog, Me, Gn) and six bilateral dental landmarks (UI, LI, U6L/R, L6L/R), as comprehensively tabulated in Table A.1.

Table A.1. Landmark Selection and Definition [1]

| **Landmarks** | | **Definition** |
| --- | --- | --- |
| **Bone tissue** | | |
| Single | N, nasion | The midpoint of the frontonasal suture |
|  | Ba, basion | The most anterior point of the great foramen (foramen magnum) |
|  | S, sella | The center of the hypophyseal fossa (sella turcica) |
|  | ANS, anterior nasal spine | The most anterior midpoint of the anterior nasal spine of the maxilla |
|  | PNS, posterior nasal spine | The most posterior midpoint of the posterior nasal spine of the palatine bone |
|  | A, subspinale | The point of maximum concavity in the midline of the alveolar process of the maxilla |
|  | B, supramental | The point of maximum concavity in the midline of the alveolar process of the mandible |
|  | Pog, pogonion | The most anterior midpoint of the chin on the outline of the mandibular symphysis |
|  | Me, menton | The most inferior midpoint of the chin on the outline of the mandibular symphysis |
|  | Gn, gnathion | The midpoint halfway between Pog and Me |
| Paired | Or, orbitale | The most inferior point of each infraorbital rim |
|  | Po, porion | The most superior point of each external acoustic meatus |
|  | Go, gonion | The point at each mandibular angle that is defined by dropping a perpendicular from the intersection point of the tangent lines to the posterior margin of the mandibular vertical ramus and inferior margin of the mandibular body or horizontal ramus |
|  | Co, condylion | The most postero-superior point of each mandibular condyle in the sagittal plane |
|  | Pmp, posterior maxillary point | The point of maximum concavity of the posterior border of the palatine bone in the horizontal plane at both sides |
|  | Zy, zygion | The most lateral point on the outline of each zygomatic arch |
|  | Fz, frontozygomatic | The most medial and anterior point of each frontozygomatic suture at the lateral orbital rim |
|  | Mx, jugal process | The intersection of the zygomatic process of the maxilla and the alveolar process |
| **Dental tissue** | | |
| Single | UI, upper incisor | The most midpoint of the crown tip of the right upper central incisor |
|  | LI, lower incisor | The most midpoint of the crown tip of the right lower central incisor |
| Pair | U6, upper molar cusp | The most inferior point of the mesial cusp of the crown of each first upper molar |
|  | L6, lower molar cusp | The most superior point of the mesial cusp of the crown of each first lower molar |
| **Soft tissue** | | |
| Single | Ns, nasion of soft tissue | The midpoint on the soft tissue contour of the nasal root base at the frontonasal suture level |
|  | Prn, pronasale | The most anterior midpoint of the nasal tip |
|  | Sn, subnasale | The midpoint on the nasolabial soft tissue contour between the columella crest and the upper lip |
|  | Ls, labrale superius | The midpoint of the vermilion line of the upper lip |
|  | Lis, labrale inferius | The midpoint of the vermilion line of the lower lip |
|  | Si, mentolabial sulcus | The most posterior midpoint on the labiomental soft tissue contour that defines the border between the lower lip and the chin |
|  | Pogs, pogonion of soft tissue | The most anterior midpoint of the chin |
|  | Mes, soft tissue menton | The most inferior midpoint of the chin |
|  | Gns, gnathion of soft tissue | The midpoint halfway between Pogs and Mes |

**1.2 Model training process**

1.2.1 Data Preprocessing

CT images for training underwent random cropping and scaling to augment the data, enabling network training independent of image size and position.

1. Random Scaling

A random scaling factor R (R­_c (number of channels)_, R_w (width)_, and R_h (height)_) was determined, where R = random (R_min_, R_max_). To ensure minimal resolution loss, R_min_ was set to 0.6. The size of the randomly cropped 3D image was defined as S (S_c_, S_w_, S_h_), and the landmark area was L (L_c_, L_w_, L_h_). To ensure that the landmark area remained entirely within the 3D image after cropping, the maximum scaling factor, R_max_, was set to S/L. Therefore, R = random (0.6, S/L). After generating a random scaling factor R, the 3D image was scaled according to this factor. The landmark coordinates x (x_c_, x_w_, x_h_) were scaled to obtain the new scaled landmark coordinates x’, which were calculated as x’=x×R.

1. Random Cropping

A random cropping box was determined using its top-left corner coordinates c (c­_c_, c_w_, c_h_) in the 3D image. The coordinates were randomly set to c = (c_min_, c_max_). The minimum boundary point of the cropping box must not be less than 0, and the maximum boundary point must not exceed the maximum boundary point of the landmark area L_max_ (L_maxc_, L_maxw_, L_maxh_); that is, c_min_=max (0, L_max_-L). The maximum boundary point of the cropping box must not exceed the size of the 3D image IS (IS_c_, IS_w_, IS_h_), and the minimum boundary point must not be greater than the minimum boundary point of the landmark area Lmin (L_minc_, L_minw_, L_minh_); that is, c_min_=max (IS-S, L_min_). Thus, c = random (max (0, L_max_-L), max (IS-S, L_min_)). The size of the cropping box, and thus the size of the image input to the model, was set to [128, 128, 64]. The image was scaled using different ratios in each dimension to calculate the minimum cuboid area containing the coordinates. A random cropping box position was selected such that all landmarks were within the box, thus completing the cropping operation. The coordinates of all corresponding landmarks in the image were adjusted by subtracting the top-left corner point of the cropping box to obtain the new coordinates x’’ of the landmarks in the cropped image, calculated as follows: x’’= x’-c.

(3) Voxel Normalization

To accelerate the convergence of the network, voxel normalization is performed on the image voxels, adjusting the voxels to the range [0, 1].

(4) Gaussian heat map.

Gaussian functions were used to transform the coordinates of each landmark into corresponding Gaussian heat maps to assist in network training. For the landmark coordinates (c_L_, w_L_, h_L_), the value of the corresponding Gaussian heatmap was

$G\left( c,w,h \right)=e^{-\frac{\left( c-c_{L} \right)^{2}+\left( h-h_{L} \right)^{2}+\left( h-h_{L} \right)^{2}}{2\sigma^{2}}}$（σ is set to 20）.

1.2.2 Model Training

During each round of model training, the image was input to the model after preprocessing. The model then computes and outputs predicted Gaussian heat maps. The loss between the predicted and true Gaussian heat maps of the network was calculated using a loss function. A loss function is a mathematical function that measures the degree of deviation between the predictions of a model and the actual values. Mean squared error (MSE) was used to represent this, and the loss function for this model was as follows:

$MSE=\frac{1}{m}\sum_{i=1}^{m} {(y_{i}-f\left( x_{i} \right))}^{2}$

The obtained loss value is used to calculate the gradients for each weight parameter through backpropagation. An Adaptive Moment Estimation (Adam) optimizer was used to optimize the model weights. This process continually refines the predicted Gaussian heat maps generated by the model during training to fit the true Gaussian heat maps, thereby achieving an optimal automatic landmarking effect.

1.2.3 Model Validation

To identify the optimal model, hyperparameters, such as the learning rate and number of iterations, were adjusted to train multiple models with different parameters. The loss of each model on the validation set was calculated, and the model with the smallest loss was selected as the optimal model.

1.2.4 Model Testing

During the testing phase, data augmentation was no longer performed. The main steps of model training and testing are illustrated in main text Figure 1.

**1.3 Selection of network backbone**

To obtain baseline data for three-dimensional skull landmarking model, 3D U-Net, V-Net (Zhao et al. 2022), FC-DenseNet (Chim et al. 2019), and Hourglass (Huang Y and Huang H 2023) were selected as CNN models for automatic landmarking of skull CT scans.

**1.4** **Definition of the metrics**

**Mean Radial Error, MRE**

$$MRE=\frac{\sum_{i=1}^{N} R_{i}}{N}$$

$R=\sqrt{\Delta x^{2}+{\Delta y}^{2}+{\Delta z}^{2}}$ ）

R: radial difference; N: number of samples; △x is the horizontal distance between automatic and gold standard landmark positions. y denotes the sagittal distance. where z denotes vertical distance.

**Standard Deviation, SD**

$$SD=\sqrt{\frac{\sum_{i=1}^{N} \left( R_{i}-MRE \right)^{2}}{N-1}}$$

**Success Detection Rate, SDR _2mm、3mm、4mm_**

$${SDR}_{2mm、3mm、4mm}=\frac{Number of detections within 2mm, 3mm, and 4mm}{Total number of detections}$$

**Dental Landmark Accuracy, DL-ACC**

$$DL-ACC=\frac{TP+TN}{TP+FP+TN+FN}$$

**Dental Landmark Precision, DL-PRE**

$$DL-PRE=\frac{TP}{TP+FP}$$

TP: Landmark present in both manual and automatic detection.

TN: Landmark absent in both manual and automatic detection.

FN: Landmark present in manual but absent in automatic detection.

FP: Landmark absent in manual but present in automatic detection.

**Intraclass Correlation Coefficient，ICC**

$$ICC=\frac{{MS}_{A}-{MS}_{e}}{{MS}_{A} +\left（ n-1 \right）{MS}_{e}}$$

MS_A_ stands for the mean square among observers, MS_e_ stands for the mean square within observers, and n represents the number of repeated measurements (n=2 in this study).

Table A.2 Relationship between Intraclass Correlation Coefficient (ICC) and degree of consistency [2]

| ICC | Degree of reliability |
| --- | --- |
| less than 0.5 | Poor reliability |
| between 0.5 and 0.75 | Moderate reliability |
| between 0.75 and 0.9 | Good reliability |
| greater than 0.90 | Excellent reliability |
| less than 0.5 | Poor reliability |

**2 Results**

**2.1 Reference observers consistency in landmarking**

Two reference observers with different backgrounds annotated the landmarks, which were reviewed by a chief physician. After a two-week gap, the annotators repeated the process. The landmarking errors on the x-, y-, and z-axes from the two sessions were analyzed to test the internal consistency of senior oral and maxillofacial surgeons and senior orthodontists, showing high internal consistency (ICC=0.965, CI: 0.941–0.981).

Table A.3. ICC assessment by reference observers

| **Landmarks** | **Intraclass ICC** | | | | | | **Interclass ICC** | | |
| --- | --- | --- | --- | --- | --- | --- | --- | --- | --- |
|  | The senior maxillofacial surgeon | | | The senior orthodontist | | |  |  |  |
|  | **X** **-axis** | **Y** **-axis** | **Z** **-axis** | **X** **-axis** | **Y** **-axis** | **Z** **-axis** | **X** **-axis** | **Y** **-axis** | **Z** **-axis** |
| **N** | 0.976 | 0.973 | 0.994 | 0.974 | 0.995 | 0.970 | 0.968 | 0.970 | 0.966 |
| **Ba** | 0.977 | 0.976 | 0.986 | 0.987 | 0.974 | 0.977 | 0.977 | 0.961 | 0.972 |
| **S** | 0.980 | 0.975 | 0.971 | 0.976 | 0.970 | 0.996 | 0.969 | 0.971 | 0.963 |
| **ANS** | 0.977 | 0.972 | 0.996 | 0.985 | 0.972 | 0.977 | 0.974 | 0.972 | 0.962 |
| **PNS** | 0.971 | 0.976 | 0.979 | 0.981 | 0.987 | 0.994 | 0.971 | 0.968 | 0.966 |
| **A** | 0.983 | 0.990 | 0.994 | 0.989 | 0.989 | 0.974 | 0.961 | 0.973 | 0.972 |
| **B** | 0.980 | 0.971 | 0.995 | 0.972 | 0.984 | 0.989 | 0.955 | 0.969 | 0.967 |
| **Pog** | 0.982 | 0.990 | 0.977 | 0.993 | 0.991 | 0.987 | 0.964 | 0.977 | 0.973 |
| **Me** | 0.973 | 0.974 | 0.977 | 0.991 | 0.992 | 0.982 | 0.972 | 0.982 | 0.972 |
| **Gn** | 0.974 | 0.977 | 0.971 | 0.984 | 0.985 | 0.972 | 0.961 | 0.965 | 0.986 |
| **UI** | 0.971 | 0.990 | 0.992 | 0.983 | 0.987 | 0.990 | 0.977 | 0.975 | 0.976 |
| **LI** | 0.974 | 0.984 | 0.976 | 0.993 | 0.996 | 0.991 | 0.961 | 0.960 | 0.964 |
| **OrL** | 0.974 | 0.978 | 0.982 | 0.979 | 0.995 | 0.978 | 0.965 | 0.966 | 0.975 |
| **OrR** | 0.970 | 0.970 | 0.990 | 0.970 | 0.972 | 0.972 | 0.962 | 0.972 | 0.975 |
| **GoL** | 0.976 | 0.971 | 0.994 | 0.972 | 0.990 | 0.974 | 0.961 | 0.963 | 0.962 |
| **GoR** | 0.978 | 0.978 | 0.982 | 0.970 | 0.986 | 0.981 | 0.963 | 0.962 | 0.966 |
| **PoL** | 0.972 | 0.996 | 0.975 | 0.974 | 0.977 | 0.984 | 0.975 | 0.976 | 0.971 |
| **PoR** | 0.974 | 0.970 | 0.990 | 0.989 | 0.971 | 0.974 | 0.977 | 0.972 | 0.976 |
| **CoL** | 0.988 | 0.975 | 0.978 | 0.970 | 0.975 | 0.996 | 0.953 | 0.960 | 0.964 |
| **CoR** | 0.974 | 0.996 | 0.970 | 0.974 | 0.996 | 0.970 | 0.952 | 0.953 | 0.962 |
| **PmpL** | 0.972 | 0.972 | 0.995 | 0.972 | 0.972 | 0.995 | 0.963 | 0.960 | 0.964 |
| **PmpR** | 0.978 | 0.974 | 0.980 | 0.977 | 0.987 | 0.979 | 0.962 | 0.973 | 0.972 |
| **ZyL** | 0.995 | 0.989 | 0.981 | 0.992 | 0.993 | 0.972 | 0.951 | 0.972 | 0.967 |
| **ZyR** | 0.975 | 0.996 | 0.990 | 0.991 | 0.979 | 0.994 | 0.961 | 0.969 | 0.968 |
| **FzL** | 0.997 | 0.972 | 0.992 | 0.995 | 0.971 | 0.990 | 0.971 | 0.968 | 0.976 |
| **FzR** | 0.978 | 0.981 | 0.997 | 0.989 | 0.982 | 0.972 | 0.971 | 0.973 | 0.972 |
| **MxL** | 0.975 | 0.976 | 0.970 | 0.974 | 0.990 | 0.982 | 0.975 | 0.969 | 0.967 |
| **MxR** | 0.976 | 0.989 | 0.970 | 0.977 | 0.991 | 0.977 | 0.971 | 0.968 | 0.966 |
| **U6L** | 0.986 | 0.989 | 0.979 | 0.991 | 0.977 | 0.972 | 0.962 | 0.970 | 0.971 |
| **U6R** | 0.986 | 0.973 | 0.974 | 0.970 | 0.986 | 0.988 | 0.955 | 0.964 | 0.962 |
| **L6L** | 0.973 | 0.979 | 0.980 | 0.987 | 0.993 | 0.977 | 0.975 | 0.971 | 0.963 |
| **L6R** | 0.995 | 0.972 | 0.996 | 0.982 | 0.984 | 0.995 | 0.972 | 0.963 | 0.962 |
| **Ns** | 0.976 | 0.983 | 0.970 | 0.995 | 0.970 | 0.972 | 0.964 | 0.971 | 0.972 |
| **Prn** | 0.972 | 0.974 | 0.979 | 0.995 | 0.981 | 0.992 | 0.956 | 0.964 | 0.967 |
| **Sn** | 0.979 | 0.997 | 0.980 | 0.988 | 0.972 | 0.992 | 0.963 | 0.962 | 0.965 |
| **Ls** | 0.977 | 0.979 | 0.983 | 0.981 | 0.984 | 0.972 | 0.975 | 0.971 | 0.974 |
| **Lis** | 0.973 | 0.974 | 0.988 | 0.972 | 0.974 | 0.991 | 0.972 | 0.974 | 0.976 |
| **Si** | 0.970 | 0.972 | 0.984 | 0.975 | 0.987 | 0.986 | 0.961 | 0.960 | 0.964 |
| **Pogs** | 0.988 | 0.970 | 0.972 | 0.978 | 0.986 | 0.986 | 0.958 | 0.963 | 0.962 |
| **Mes** | 0.984 | 0.995 | 0.973 | 0.978 | 0.972 | 0.977 | 0.962 | 0.948 | 0.975 |
| **Gns** | 0.983 | 0.972 | 0.994 | 0.991 | 0.988 | 0.989 | 0.951 | 0.962 | 0.967 |

Note: ICC = intraclass correlation coefficient

**2.2 Model Hyperparameters and model training loss graph**

Figure A.1 shows the training loss graphs for the automatic landmarking model when utilizing the four different network backbones.

| 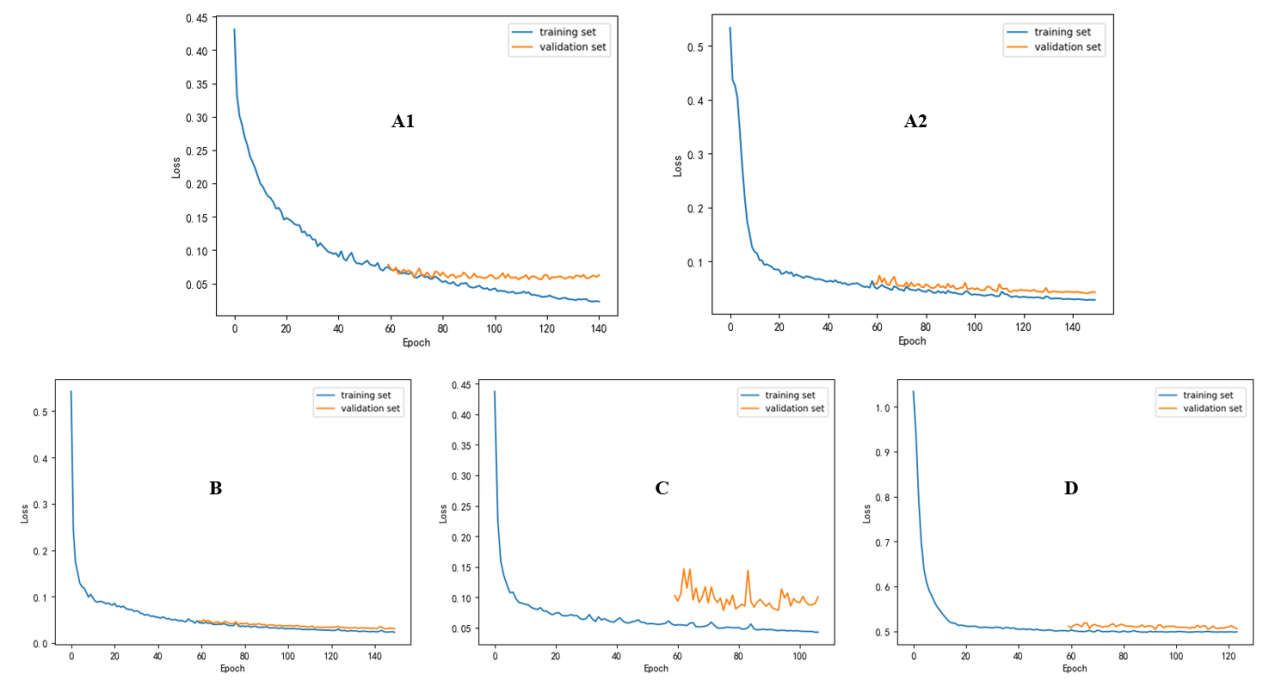 |
| --- |

Figure A.1. Model Training Loss Graph

*A1* is the CBCT 3D U-Net, *A2* is the SCT 3D U-Net, *B* is the SCT V-Net, *C* is the SCT FC-DenseNet, and *D* is the SCT Hourglass. The x-axis represents the training iterations, and the y-axis represents the training loss. In *A1, A2, B, D,* the validation and training losses were similar, suggesting a good generalization. In *C*, the discrepancy indicates poor generalization.

**2.3 Results of the four neural networks**

Table A.4. MRE of landmarks in SCT's test set for 3D U-Net, V-Net, FC-DenseNet, Hourglass (MRE ± SD, mm)

| **Landmarks** | **3D U-Net** | **V-Net** | **FC-DenseNet** | **Hourglass** |
| --- | --- | --- | --- | --- |
| **N** | 1.122±0.536 | 1.464±0.884 | 1.392±0.710 | 1.669±0.722 |
| **Ba** | 1.248±0.562 | 1.496±0.780 | 1.177±0.502 | 1.350±0.468 |
| **S** | 1.237±0.554 | 1.491±0.778 | 1.175±0.500 | 1.348±0.463 |
| **ANS** | 1.375±0.863 | 1.412±1.050 | 1.457±0.551 | 1.651±1.751 |
| **PNS** | 0.956±0.806 | 1.197±1.032 | 1.017±0.376 | 0.926±0.375 |
| **A** | 1.191±0.687 | 1.076±0.721 | 1.334±0.775 | 1.204±1.044 |
| **B** | 1.077±0.599 | 1.280±0.895 | 1.894±0.961 | 1.085±0.562 |
| **Pog** | 0.909±0.797 | 1.172±1.473 | 1.171±0.548 | 0.964±0.553 |
| **Me** | 0.939±0.514 | 0.880±0.529 | 1.131±0.637 | 1.098±0.557 |
| **Gn** | 0.714±0.443 | 1.712±0.488 | 0.961±0.600 | 0.933±0.521 |
| **UI** | 1.337±0.588 | 1.416±0.955 | 1.554±0.626 | 1.264±0.573 |
| **LI** | 1.223±0.688 | 1.999±0.853 | 1.810±0.791 | 1.192±0.549 |
| **OrL** | 0.807±0.452 | 1.052±0.605 | 0.870±0.355 | 0.782±0.406 |
| **OrR** | 0.771±0.363 | 1.012±0.670 | 0.873±0.394 | 1.049±0.447 |
| **GoL** | 0.981±0.556 | 1.176±0.882 | 1.435±0.824 | 1.430±0.709 |
| **GoR** | 0.957±0.530 | 1.230±0.915 | 1.581±0.991 | 1.456±0.915 |
| **PoL** | 1.034±0.394 | 1.135±0.564 | 1.011±0.466 | 1.147±0.512 |
| **PoR** | 1.068±0.592 | 1.210±0.905 | 1.170±0.569 | 1.351±0.603 |
| **CoL** | 0.975±0.488 | 1.160±0.712 | 1.225±0.607 | 1.245±0.601 |
| **CoR** | 1.108±0.707 | 1.203±0.755 | 1.250±0.702 | 1.305±0.625 |
| **PmpL** | 0.825±0.473 | 1.156±0.762 | 0.980±0.323 | 0.918±0.407 |
| **PmpR** | 0.772±0.497 | 1.049±0.748 | 0.898±0.433 | 0.852±0.337 |
| **ZyL** | 1.303±0.581 | 1.201±0.580 | 1.093±0.411 | 1.275±0.657 |
| **ZyR** | 1.354±0.986 | 1.105±1.046 | 1.058±0.586 | 1.330±0.505 |
| **FzL** | 1.097±0.547 | 1.231±0.610 | 1.373±0.414 | 1.572±0.654 |
| **FzR** | 1.120±0.505 | 1.370±0.760 | 1.750±0.474 | 1.621±0.718 |
| **MxL** | 0.954±0.529 | 0.923±0.521 | 1.350±0.651 | 1.082±0.589 |
| **MxR** | 1.115±0.703 | 1.107±0.648 | 1.364±0.508 | 1.318±0.604 |
| **U6L** | 0.944±0.612 | 1.083±0.900 | 1.256±0.573 | 1.112±0.603 |
| **U6R** | 1.025±0.649 | 1.221±0.348 | 1.478±0.673 | 1.467±0.797 |
| **L6L** | 1.102±0.703 | 1.142±0.826 | 1.409±0.679 | 1.490±0.846 |
| **L6R** | 1.052±0.548 | 1.141±0.829 | 1.227±0.637 | 1.374±0.892 |
| **Ns** | 1.132±0.855 | 1.169±0.567 | 1.589±0.555 | 0.925±0.374 |
| **Prn** | 1.039±0.923 | 1.410±0.655 | 1.105±0.485 | 1.104±0.485 |
| **Sn** | 1.206±0.908 | 1.956±0.613 | 1.029±0.487 | 1.167±0.633 |
| **Ls** | 1.439±0.868 | 1.572±1.029 | 1.400±0.885 | 1.552±0.767 |
| **Lis** | 1.821±0.911 | 1.405±1.112 | 1.385±0.803 | 1.222±0.464 |
| **Si** | 1.121±0.587 | 1.357±0.764 | 1.349±0.642 | 1.339±0.494 |
| **Pogs** | 1.006±0.522 | 2.384±0.998 | 2.138±2.785 | 1.575±1.743 |
| **Mes** | 1.362±0.602 | 2.604±0.883 | 3.184±5.592 | 2.635±3.914 |
| **Gns** | 1.072±0.531 | 1.503±0.581 | 2.486±4.616 | 2.349±4.976 |
| **Average** | 1.091±0.306 | 1.335±0.358 | 1.381±0.884 | 1.319±0.847 |

Note: MRE = mean radial error, SD = standard deviation

Table A.5. MRE of the four network architectures in SCT and CBCT's test set

| **Network Framework** | **SCT MRE±SD，mm** | **CBCT MRE±SD，mm** |
| --- | --- | --- |
| Modified 3D U-Net | 1.091±0.306 | 0.924±0.830 |
| V-Net | 1.335±0.358 | 1.553±1.358 |
| FC-DenseNet | 1.381±0.884 | 1.681±1.109 |
| Hourglass | 1.319±0.847 | 1.585±1.047 |

Note: MRE (Mean Radial Error), SD (Standard Deviation)

From the results in Table A.4 and A.5, it is evident that the 3D U-Net has the smallest MRE among the four network skeletons (SCT: 1.091±0.306 mm, CBCT: 0.924±0.83 mm). The MRE of 3D U-Net was compared with those of V-Net, FC-DenseNet, and Hourglass using the Mann–Whitney U test, as shown in Figure A.2, indicating a statistically significant difference in MRE between 3D U-Net and the other three network skeletons. The analysis indicates that when 3D U-Net was used as the model network, it had the highest accuracy.

| 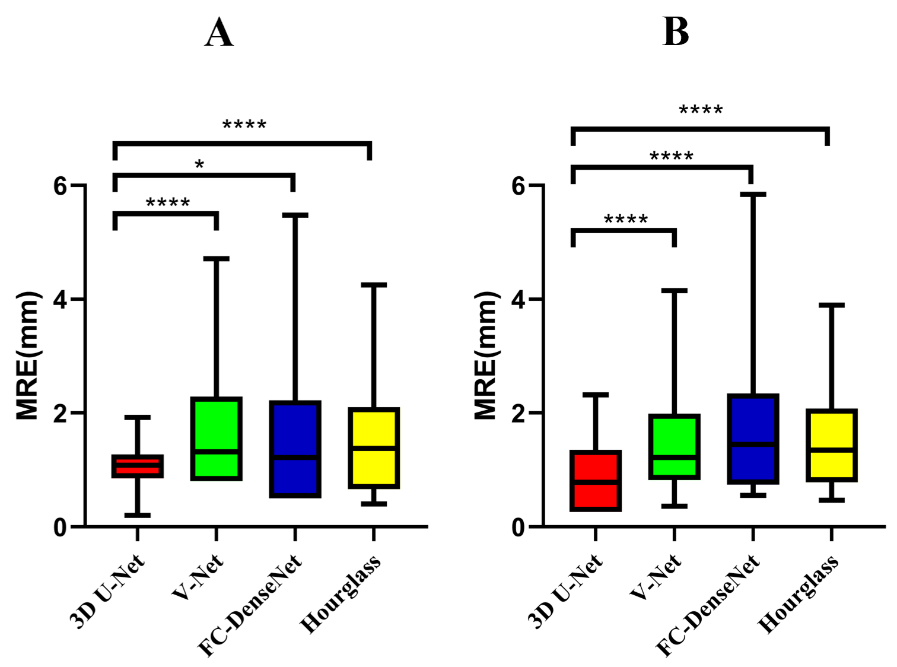 |
| --- |

Figure A.2. Boxplot of landmark error statistical tests for various network architectures

*A:* SCT network architecture test outcomes; *B:* CBCT network architecture test outcomes. **** denotes P < 0.0001, * denotes P < 0.05. Note: MRE (mean radial error)

**2.4 Landmark positioning results in the external test set**

Table A.6. MRE and SDR of landmarks in SCT's external test set

| **Landmarks** | **MRE±SD，mm** | **SDR，%** | | |
| --- | --- | --- | --- | --- |
|  |  | **2mm** | **3mm** | **4mm** |
| **N** | 1.454±0.835 | 91.6 | 99.1 | 99.1 |
| **Ba** | 1.165±0.593 | 97.5 | 99.1 | 100 |
| **S** | 1.161±0.595 | 97.5 | 99.1 | 100 |
| **ANS** | 1.156±0.739 | 93.1 | 97.4 | 99.1 |
| **PNS** | 0.804±0.509 | 98.3 | 100 | 100 |
| **A** | 1.006±0.476 | 96.8 | 100 | 100 |
| **B** | 1.187±0.736 | 89.1 | 98.3 | 100 |
| **Pog** | 1.047±0.689 | 93.3 | 99.1 | 100 |
| **Me** | 1.114±0.708 | 94.9 | 98.3 | 99.1 |
| **Gn** | 0.951±0.503 | 98.3 | 100 | 100 |
| **UI** | 1.374±1.215 | 89.0 | 98.3 | 99.1 |
| **LI** | 1.745±0.849 | 69.8 | 93.9 | 99.1 |
| **OrL** | 0.923±0.499 | 99.1 | 100 | 100 |
| **OrR** | 0.924±0.499 | 99.1 | 100 | 100 |
| **GoL** | 1.229±1.366 | 88.3 | 99.1 | 99.1 |
| **GoR** | 1.261±0.922 | 90.0 | 95.8 | 99.1 |
| **PoL** | 1.193±0.674 | 94.1 | 99.1 | 99.1 |
| **PoR** | 1.151±0.582 | 97.5 | 99.1 | 100 |
| **CoL** | 1.236±0.589 | 93.1 | 100 | 100 |
| **CoR** | 1.354±0.794 | 95.0 | 98.3 | 99.1 |
| **PmpL** | 0.860±0.718 | 99.1 | 99.1 | 99.1 |
| **PmpR** | 0.868±0.558 | 98.3 | 99.1 | 100 |
| **ZyL** | 1.359±0.679 | 90.0 | 98.3 | 99.1 |
| **ZyR** | 1.264±0.720 | 90.7 | 98.3 | 99.1 |
| **FzL** | 1.241±0.705 | 90.0 | 100 | 100 |
| **FzR** | 1.416±0.758 | 80.8 | 100 | 100 |
| **MxL** | 1.187±0.623 | 95.7 | 99.1 | 100 |
| **MxR** | 1.237±0.599 | 93.3 | 99.1 | 100 |
| **U6L** | 1.347±0.600 | 90.7 | 99.1 | 100 |
| **U6R** | 1.327±0.695 | 91.5 | 99.1 | 100 |
| **L6L** | 1.430±0.766 | 83.3 | 95.0 | 99.1 |
| **L6R** | 1.333±0.592 | 90.0 | 98.3 | 100 |
| **Ns** | 0.993±0.674 | 95.0 | 99.1 | 99.1 |
| **Prn** | 0.794±0.475 | 100 | 100 | 100 |
| **Sn** | 1.448±0.724 | 92.5 | 98.3 | 99.1 |
| **Ls** | 1.268±0.796 | 90.8 | 97.5 | 99.1 |
| **Lis** | 1.487±1.228 | 83.3 | 95.0 | 99.1 |
| **Si** | 1.365±0.650 | 91.6 | 99.1 | 100 |
| **Pogs** | 1.421±0.778 | 80.0 | 98.0 | 100 |
| **Mes** | 1.714±0.989 | 76.1 | 94.2 | 99.0 |
| **Gns** | 1.297±0.809 | 89.5 | 97.1 | 99.0 |

Note: MRE (mean radial error), SDR (success detection rate), SD (standard deviation)

Table A.7. MRE and SDR of landmarks in CBCT's external test set

| **Landmarks** | **MRE±SD, mm** | SDR，% | | |
| --- | --- | --- | --- | --- |
|  |  | **2mm** | **3mm** | **4mm** |
| **S** | 1.118±0.712 | 86.4 | 97.7 | 100 |
| **A** | 0.997±0.650 | 92.9 | 97.6 | 100 |
| **B** | 1.122±0.726 | 84.1 | 97.7 | 100 |
| **Pog** | 1.121±0.699 | 86.4 | 97.7 | 100 |
| **Me** | 0.996±0.744 | 81.8 | 97.7 | 97.7 |
| **Gn** | 1.183±0.718 | 84.1 | 97.7 | 100 |
| **UI** | 0.922±0.813 | 83.7 | 93.6 | 97.7 |
| **LI** | 0.917±0.732 | 88.1 | 94.7 | 100 |
| **GoL** | 1.275±0.585 | 88.1 | 97.6 | 100 |
| **GoR** | 1.491±0.741 | 80.5 | 88.1 | 95.2 |
| **U6L** | 0.946±0.832 | 84.1 | 93.2 | 100 |
| **U6R** | 0.939±1.181 | 84.1 | 93.2 | 97.7 |
| **L6L** | 1.008±1.184 | 80.3 | 93.2 | 93.2 |
| **L6R** | 1.006±1.082 | 88.6 | 93.2 | 95.5 |

Note: MRE (mean radial error), SD (standard deviation), SDR (success detection rate)

**2.5** **The landmark's positioning errors along each coordinate axis**

The MRE for landmarks on three-dimensional images were composed of their respective errors along the x-, y-, and z-axes. The average positioning errors of the landmarks along each coordinate axis in the test set were calculated, and listed in Table A.8 and Figure A.3.

Table A.8. SCT landmark localization errors on each coordinate axis

| **Landmarks** | **△x±SD, mm** | **△y±SD, mm** | **△z±SD , mm** |
| --- | --- | --- | --- |
| **N** | 0.58±0.36 | 1.07±0.84 | 0.79±1.02 |
| **Ba** | 0.41±0.24 | 0.73±0.61 | 0.82±0.56 |
| **S** | 0.39±0.22 | 0.85±0.68 | 0.81±0.56 |
| **ANS** | 0.47±0.30 | 0.64±0.49 | 0.87±0.68 |
| **PNS** | 0.21±0.10 | 0.33±0.26 | 0.74±0.55 |
| **A** | 0.38±0.14 | 0.28±0.16 | 0.89±0.72 |
| **B** | 0.59±0.49 | 0.78±0.72 | 0.68±0.66 |
| **Pog** | 0.48±0.33 | 0.59±0.54 | 0.71±0.47 |
| **Me** | 0.49±0.41 | 0.39±0.28 | 0.90±0.85 |
| **Gn** | 0.64±0.52 | 0.51±0.44 | 0.47±0.65 |
| **UI** | 1.14±1.13 | 0.53±0.51 | 0.56±0.82 |
| **LI** | 0.64±0.53 | 0.70±0.65 | 1.39±1.02 |
| **OrL** | 0.75±0.51 | 0.38±0.26 | 0.41±0.28 |
| **OrR** | 0.76±0.55 | 0.38±0.27 | 0.40±0.25 |
| **GoL** | 0.58±0.57 | 0.64±0.61 | 0.88±0.91 |
| **GoR** | 0.29±0.26 | 0.37±0.31 | 1.19±0.97 |
| **PoL** | 0.67±0.66 | 0.47±0.59 | 0.89±0.70 |
| **PoR** | 0.65±0.59 | 0.43±0.60 | 0.89±0.67 |
| **CoL** | 0.89±0.78 | 0.60±0.46 | 0.59±0.47 |
| **CoR** | 0.92±0.89 | 0.61±0.54 | 0.61±0.50 |
| **PmpL** | 0.53±0.68 | 0.30±0.28 | 0.59±0.51 |
| **PmpR** | 0.53±0.46 | 0.31±0.28 | 0.61±0.52 |
| **ZyL** | 1.21±0.76 | 0.46±0.36 | 0.52±0.71 |
| **ZyR** | 0.55±0.31 | 0.64±0.45 | 0.91±0.93 |
| **FzL** | 0.47±0.38 | 0.91±0.71 | 0.73±0.60 |
| **FzR** | 0.56±0.46 | 1.13±0.89 | 0.72±0.66 |
| **MxL** | 0.57±0.47 | 0.63±0.46 | 0.79±0.62 |
| **MxR** | 0.65±0.50 | 0.59±0.44 | 0.87±0.86 |
| **U6L** | 0.80±0.46 | 0.54±0.30 | 0.91±0.96 |
| **U6R** | 0.78±0.67 | 0.51±0.31 | 0.90±0.75 |
| **L6L** | 0.68±0.54 | 0.41±0.28 | 1.18±0.85 |
| **L6R** | 0.70±0.49 | 0.38±0.26 | 1.16±0.73 |
| **Ns** | 0.36±0.25 | 0.52±0.51 | 0.79±0.77 |
| **Prn** | 0.47±0.30 | 0.61±0.49 | 0.30±0.22 |
| **Sn** | 0.46±0.26 | 0.41±0.24 | 1.29±0.81 |
| **Ls** | 0.42±0.27 | 0.55±0.52 | 1.06±0.88 |
| **Lis** | 0.47±0.38 | 1.19±1.01 | 0.76±0.77 |
| **Si** | 0.56±0.26 | 0.93±0.80 | 0.88±0.40 |
| **Pogs** | 0.53±0.49 | 0.86±0.66 | 1.01±0.81 |
| **Mes** | 0.57±0.33 | 0.64±0.41 | 1.49±1.26 |
| **Gns** | 0.53±0.51 | 0.49±0.33 | 1.07±1.28 |
| **Average** | 0.81±0.23 | 0.81±0.69 | 1.14±0.30 |


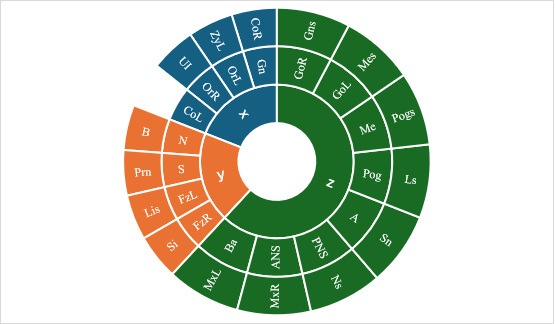
Figure A.3. The landmarks with the largest positioning errors on different axes.

**2.6 Other visualization presentations**

Red landmarks represent the reference standard, and green ones are the predicted landmarks.

| 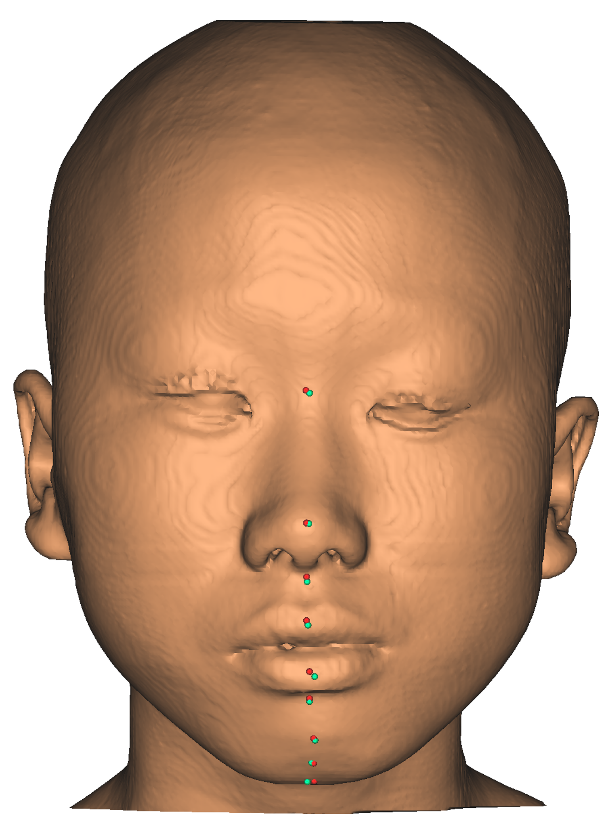 |
| --- |

Figure A.4. List landmarks on a 3D plane across soft tissues

This study incorporated nine facial soft tissue landmarks in the SCT that are vital for symmetry assessment. Influenced by factors like body position and muscle pull, these landmarks are less distinct and more difficult to automatically localize than hard tissues. Despite a slightly higher MRE than that of the bones, this study shows the potential for automated localization of maxillofacial soft tissue landmarks.

| 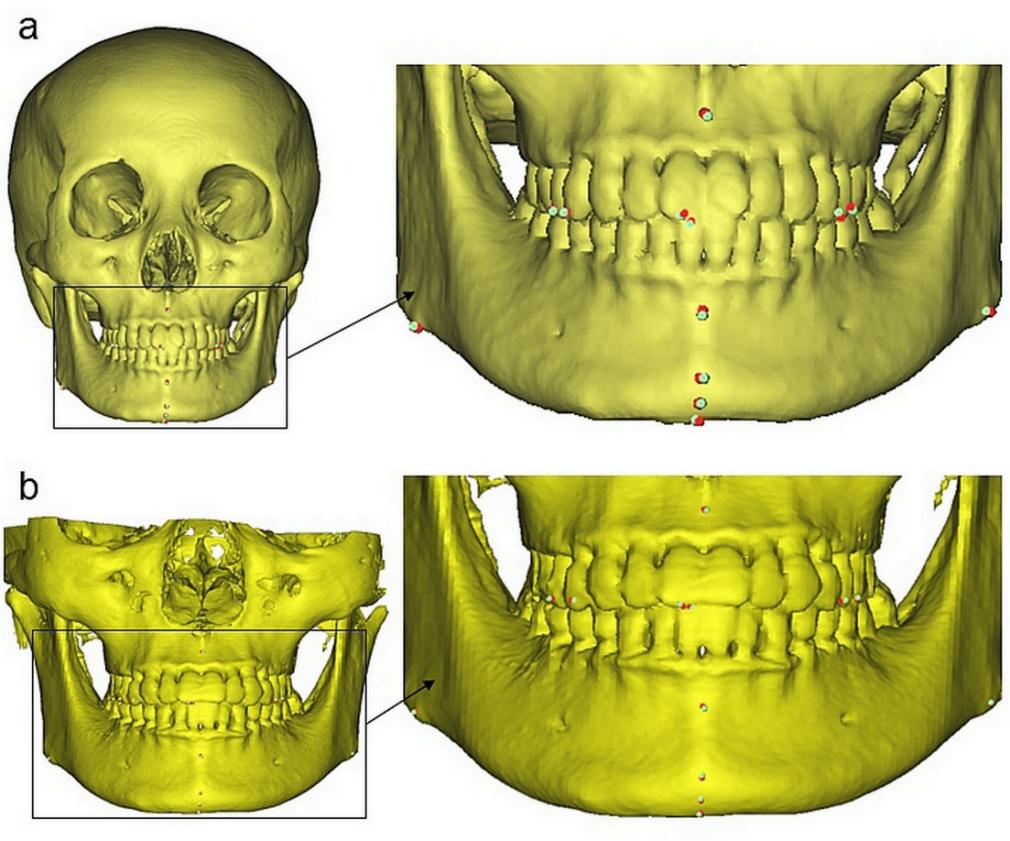 |
| --- |

Figure A.5. Comparative localization of landmarks using SCT and CBCT at different time periods. A 40-year-old woman underwent CBCT for dental issues in 2021 and SCT for TMJ issues in 2022. 3D landmarking from SCT (panel a) showed lower MREs (1.221 mm) than CBCT (panel b, 1.334 mm) for bone landmarks, except for A. CBCT had lower errors for dental landmarks, except for L6L. The largest MRE difference was for GoR (1.158 mm) and the smallest for A (0.006 mm).

**References**

1. Swennen GR, Schutyser FA, Hausamen JE. 2005. Three-dimensional cephalometry: a color atlas and manual. Berlin Heidelberg: Springer Science & Business Media.

2. Koo TK, Li MY. A Guideline of Selecting and Reporting Intraclass Correlation Coefficients for Reliability Research. J Chiropr Med. 2016 Jun;15(2):155-63. doi: 10.1016/j.jcm.2016.02.012. Epub 2016 Mar 31. Erratum in: J Chiropr Med. 2017 Dec;16(4):346. doi: 10.1016/j.jcm.2017.10.001. PMID: 27330520; PMCID: PMC4913118.
